# High dietary oleic acid in olive oil‐supplemented diet enhanced omega‐3 fatty acid in blood plasma of rats

**DOI:** 10.1002/fsn3.1644

**Published:** 2020-05-19

**Authors:** Kim Margarette C. Nogoy, Hyoun Ju Kim, Yehyun Lee, Yan Zhang, Jia Yu, Dong Hoon Lee, Xiang Zi Li, Stephen B. Smith, Hyun A Seong, Seong Ho Choi

**Affiliations:** ^1^ Department of Animal Science Chungbuk National University Cheongju City South Korea; ^2^ National Institute of Animal Science Wanju‐gun South Korea; ^3^ Department of Biosystems Engineering Chungbuk National University Cheongju City South Korea; ^4^ Co‐Innovation Center of Beef Cattle Science and Industry Technology Yanbian University Yanji China; ^5^ Department of Animal Science Texas A&M University College Station TX USA; ^6^ Department of Biochemistry Chungbuk National University Cheongju City South Korea

**Keywords:** cardiovascular disease, Hanwoo beef fat, oleic acid, olive oil, omega‐3, polyunsaturated fatty acids

## Abstract

This study was conducted to investigate the effect of dietary oleic acid in olive oil‐supplemented diets on the blood lipid profile and fatty acid composition in blood plasma and adipose tissue of rats. A total of 60 Sprague Dawley rats with mean body weight of 249 g ± 3.04 g were equally divided into three diet groups: control (CON) contained 10% coconut oil, olive50 contained 5% coconut oil and 5% olive oil, and olive100 contained 10% olive oil. Oleic acid (OA) level was highest in olive100 followed by the olive50 and control. The final body weight (BW) of the rats was significantly affected by the intake of OA, in which rats fed olive100 had the lowest final BW, which signified that OA could be associated with weight loss. Olive oil intake significantly increased levels of the high‐density lipoprotein cholesterol (HDL‐C) and exhibited a potential attenuation effect on the glutamic‐oxaloacetic transaminase and the glutamic‐pyruvic transaminase, and a potential role in the reduction of triglycerides in the bloodstream of the animals. In terms of fatty acid composition, significantly high OA was observed in the blood plasma and adipose tissues of rats fed olive100. Omega‐3 polyunsaturated fatty acids (PUFAs), such as linolenic (C18:3 *n*−3), eicosapentaenoic (C20:5 *n*−3), and docosahexaenoic (C22:6 *n*−3), and *n*−6 PUFA arachidonic (C20:4 *n*−6) were also significantly increased in the blood plasma of rats fed olive100. These findings suggest that the intake of dietary high OA may enhance the omega‐3 fatty acid levels in the blood plasma of rats and may have a positive effect in reducing risks to cardiovascular disease, as evidenced by weight loss, increased HDL‐C levels, and decreased TG levels in the blood plasma of experimental animals.

## Graphical abstract

We found that dietary oleic acid present in olive oil‐supplemented diet fed in rats increased the omega‐3 PUFAs such as ALA, EPA, and DHA and has a potentially positive effect in reducing CVD risks as supported by weight loss and decreased TG levels in the bloodstream of rats.

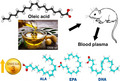

## 1 INTRODUCTION

Oleic acid (OA) is classified as a monounsaturated fatty acid (MUFA, cis‐18:1*n*−9) that is found abundantly in olive oil, representing 70%‐80% of the olive oil composition (Owen et al., 2000). It is also a nonessential fatty acid that has been described as a regulator of immune function and health and its substitution to dietary saturated fat has been described to reduce the risks of CVD by reducing blood lipids, such as cholesterol. Oleic acid has been also reported to decrease the circulating concentration of the low‐density lipoprotein (LDL) cholesterol in humans (Kwon & Choi, 2015; Melton, Amiri, Davis, & Backus, 1982; Rudel, Park, & Sawyer, 1995; Smith, 1994) and has been found to increase the beneficial high‐density lipoprotein (HDL) cholesterol concentration in blood (Gilmore et al., 2011). As per recent reports, the potential effect of OA in amelioration of a CVD risk may be associated with an improvement of the serum lipoprotein profile (HDL‐to‐LDL) in patients with hypercholesterolemia (Bemelmans et al., 2002; Zambon et al., 2000).

The protective action of the regular intake of OA on health risk parameters—such as CVD—has been mainly reported in the Mediterranean region, where diet is associated with an elevated MUFA intake in the consumption of olive oil (Martinez‐Gonzalez & Sanchez‐Villegas, 2004; Menotti et al., 1990). In South Korea, the Hanwoo beef industry has been criticized over the past decade due to high‐fat levels in the Hanwoo beef, and the association between saturated fats and obesity and cardiovascular disease (CVD). This slowed the rate of per capita consumption of the Hanwoo beef in Korea. The highly marbled Hanwoo beef contains an abundant amount of MUFA, especially oleic acid (Cho et al., 2005; Hwang & Joo, 2016, 2017; Jung, Hwang, & Joo, 2015, 2016). It also contains the main saturated fatty acids (SFAs) that are commonly present in beef intramuscular fat (IMF), such as palmitic (C16:0) and stearic acid (C18:0). These SFAs typically comprise slightly < 50% of the total lipid composition (Frank et al., 2016; Hwang & Joo, 2016, 2017). The presence of these SFAs in beef is the main cause of health concerns and associations with CVD (Bingham, Hughes, & Cross, 2002). However, the IMF in Hanwoo beef has a higher overall concentration of MUFA—predominantly oleic acid—which is considered a healthy fat (Kwon & Choi, 2015; Rudel & Sawyer, 1995; Smith, 1994). Despite the number of studies conducted on fatty acid composition of the Hanwoo beef and the related health effects of these fatty acids, many of the beef consumers continue to associate the consumption of highly marbled beef to unhealthy eating. Hence in this study, we aimed to provide a new and current evaluation of the effect of oleic acid on health‐related parameters. Specifically, we aimed to determine the growth performance, blood lipid profile, and fatty acid composition in blood plasma and adipose tissues of rats that were fed with olive oil. Our laboratory conducted two sets of experiment for this topic: The first experiment focused on the oleic acid in olive oil, and the second experiment focused on the oleic acid in the Hanwoo beef fat.

## 2 MATERIALS AND METHODS

### 2.1 Laboratory animals and treatments

Sixty (60) male Sprague‐Dawley rats (249 ± 3.04 g) were used in this experiment. The experimental animals were placed in groups of two per cage under the following controlled conditions: room temperature (22°C), 12‐hr light–dark cycle, 40%–60% relative humidity, and water ad libitum. The animals were allowed to adapt for 2 weeks in the animal room of the Chungbuk National University. After acclimation, the experimental rats were equally divided into the following three treatment groups: the control group that contained 10% coconut oil as the fat source, olive50 group that contained both 5% coconut oil and 5% olive oil as the oleic acid source, and olive100 group that contained 10% olive oil. All procedures adhered to the NIH Guidelines for Care and Use of Laboratory Animals.

### 2.2 Analytical assessment of diets

The composition of the rat diets has been shown in Table 1 and was prepared by the extrusion method. The formulation was based on the American Institute of Nutrition AIN‐76A method described by Dyets (Reeves, Nielsen, & Fahey, 1993) with slight modifications (the sugar content was reduced from 50% to 45%, and instead of corn oil, coconut and olive oil were used). All rat chows were vacuum‐packed and stored at 4°C. The same energy portion was provided to all treatments, and the total energy for all treatments was 4,152 kcal/kg. The chemical composition, including dry matter (DM), crude fat (EE), crude protein (CP), and ash, of each diet was analyzed based on the AOAC method (1995). Values have been shown in Table 1, and all treatment groups had nearly identical chemical composition. The fatty acid composition of the diets was determined by gas chromatography, as described below, and has been shown in Table 2. The CON group exhibited the highest C12:0 and C14:0 contents due to coconut oil, which mainly contains medium‐chain fatty acids. Olive50 and Olive100 exhibited higher C18:1 than that exhibited by the CON group, which implied that olive oil was a source of oleic acid (C18:1).

**TABLE 1:** Ingredient and chemical composition of three rat chow diets

Items	CON^a^, %	Olive50^b^, %	Olive100^c^ %
Ingredient			
Granular sugar	45	45	45
Casein lactic	20	20	20
Cornstarch	15	15	15
Solka Floc‐40	5	5	5
Coconut oil	10	5	‐
Olive oil	—	5	10
AIN‐76A mineral mix	3.5	3.5	3.5
AIN‐76A vitamin mix	1	1	1
DL‐methionine	0.3	0.3	0.3
Choline bitartrate	0.2	0.2	0.2
Total	100	100	100
Chemical composition			
DM^d^	99.35	99.37	99.48
EE^e^	12.37	12.16	12.56
CP^f^	15.43	15.13	16
ASH	4.05	4.05	4.16

^a^ Control.

^b^ 5% coconut oil + 5% olive oil.

^c^ 10% olive oil.

^d^ Dry matter.

^e^ Ether extract.

^f^ Crude protein.

**TABLE 2:** Fatty acid composition (g/100 g diet) of the different treatment diets

Fatty acids	Content (g/100 g diet)		
	CON^a^	Olive50^b^	Olive100^c^
C_12:0 lauric acid_	5,143	2,487	58
C_14:0 myristic acid_	2,006	964	25
C_16:0 palmitic acid_	931	1,026	1,174
C_16:1 myristoleic acid_	2	47	90
C_18:0 stearic acid_	349	367	411
C_18:1_ *_n_*_−9 oleic acid_	579	4,634	8,717
C_18:2_ *_n_*_−6 linoleic acid_	354	303	486
C_18:3_ *_n_*_−3 linolenic acid_	1	41	70
C_20:0_	9	26	43
C_20:1_	3	55	30
C_20:2_	1	3	5
C_20:3_	—	4	4
C_20:4 arachidonic acid_	—	—	—
C_20:5 eicosapentaenoic acid_	33	23	3
C_22:0_	1	7	11
C_22:1_	2	2	2
C_24:0_	94	4	5
C_24:1_	—	—	21
C_22:6 docosahexaenoic acid_	—	1	4
Total SFA^d^	8,533	4,881	1,727
Total MUFA^e^	586	4,738	8,860
Total PUFA^f^	389	375	572
Total	9,508	9,994	11,159

^a^ Control.

^b^ 5% coconut oil + 5% olive oil.

^c^ 10% olive oil.

^d^ Saturated fatty acids

^e^ Mono‐unsaturated fatty acids

^f^ Poly‐unsaturated fatty acids.

### 2.3 Determination of growth performance and organ collection

Feed intake and body weight were measured and recorded daily. Feed intake was measured by weighing food, including spilled feed, in each cage dispenser within the cage. After 4 weeks, ten experimental rats per treatment group were starved overnight and were then anesthetized with ethyl ether and sacrificed. The remaining groups of experimental animals were sacrificed after 8 weeks. Blood samples were collected via an abdominal aorta puncture, contained in a vacutainer, and were centrifuged at 1075 *g* for 20 min to collect plasma for biochemical analysis. Internal organs, such as liver, kidneys, heart, lungs, spleen, and the subcutaneous fat, were surgically removed, weighed, and immediately snap‐frozen in liquid nitrogen.

### 2.4 Biochemical analysis

Levels of triglycerides (TGs), total cholesterol (TC), high‐density lipoprotein (HDL) cholesterol, low‐density lipoprotein (LDL) cholesterol, glutamic‐oxaloacetic transaminase (GOT), and glutamic‐pyruvic transaminase (GPT) in the plasma were measured using a chemical analyzer (Adiva2021, Hitachi).

### 2.5 Fatty acid composition analysis

To analyze the fatty acid composition of the blood plasma and subcutaneous fat of rats, 1 ml of plasma and 0.3 g of the adipose tissue were isolated and placed in a 10 ml of the Folch solution (chloroform: methanol = 2:1, v/v). To quantify the fatty acid composition, 5 mg of C13:0 as an internal standard was added to each sample and homogenized for 1 min at 16,028 *g* (CH/PT3100 Polytron, Kinematica). Next, 5 ml of 0.74% KCl was added, vortexed for 1 min, and centrifuged at 1912 *g* for 30 min at 4°C. The bottom layer with the lipids and chloroform was then evaporated at 70°C using a heating block and N_2_ gas. After evaporation, the lipids were saponified with 1 ml 0.5 M KOH in methanol at 90°C for 10 min and methylated with 1 ml of 14% BF3 in methanol at 90°C for 30 min. The fatty acid methyl ester (FAME) was mixed and centrifuged with hexane and NaCl, and the top layer was evaporated to calculate the actual FAME amount and diluted again with HPLC‐grade hexane. Prepared samples were analyzed by HP6890 gas chromatography using the SPTM‐2560 fused silica capillary column (Supelco, 100 m × 0.25 mm × 0.2 μl film thickness). The inlet temperature was 270°C, and the flame ionization detector (FID) was maintained at 300°C. The oven temperature was maintained at 150°C for 10 min, programmed to increase 2°C per min until it reached 220°C, and then maintained at 220°C for 10 min. The flow rate of the column was set to 10 cm/min, and the split/splitless ratio was set to 50:1. Fatty acids were quantified using a standard mixture (Nucheck‐prep Company), and C13:0 fatty acid (Sigma‐Aldrich) was used as the internal standard. Inserts were used for GC analysis for blood plasma samples due to their low fatty acid content.

### 2.6 Statistical analysis

The results of this experiment were analyzed by the SAS GLM method (SAS Institute & Inc, 2004). The differences in feeding periods among treatments were measured. Interactions between feed periods and oleic acid intake were investigated. The difference between means was considered significant at *p* < .05.

## 3 RESULTS

### 3.1 Effects of olive oil on growth performance and weight characteristics of the internal organs

After 4 weeks of feeding, the body weight and feed intake of the experimental animals were not significantly affected by the different diets (Table 3). After 8 weeks of feeding, olive100 significantly lowered the body weight of the experimental animals, and no difference in feed intake was found between groups. The lower body weight in olive100 could not be attributed to the reduced feed intake, as the feed intake was equivalent between groups. Experimental animals that were fed with olive100 had the same lower body weight compared with that in the control diet, which implied that olive oil was associated with—but not the direct cause of—weight loss. The increase in the body weight of rats that were fed with olive50 seemed to be due to the interaction between unsaturated fatty acids in olive oil and saturated fatty acids in coconut oil. Internal organs of the experimental rats were also recorded, and no significant difference in the organ weights among the diet groups was found, except for the olive50 group with a significantly increased weight of the lungs.

**TABLE 3:** Growth performance and organ weights of experimental animals that were fed with olive oil

Items	Treatments						*SEM* ^f^	Effects			
	4 weeks			8 weeks				Period(P)	Oil(O)	P × O	
	CON^a^	Olive50^b^	Olive100^c^	CON^a^	Olive50^b^	Olive100^c^					
Growth performance										
Initial BW^d^(g)	249	244	255	252	251	247	1.439	N.S.	N.S.	N.S.
Final BW(g)	416	411	417	472	504	478	5.629	***	*	N.S.
ADG(g)^e^	5.98	5.96	5.78	3.93	4.51	4.13	0.392	***	*	N.S.
Weight gain(g)	167	167	162	220	252	231	8.104	***	*	N.S.
Feed intake(FI)/day	41.14	41.11	41.68	40.7	43.2	40.6	0.141	N.S.	N.S.	N.S.
ADG/FI	0.145	0.145	0.139	0.1	0.1	0.1	0.01	***	N.S.	N.S.
Organ weight(g)										
Liver	10.7	10.7	10.5	14.19	12.91	13.68	0.281	***	N.S.	N.S.
Kidney	2.66	2.61	2.6	2.95	2.98	2.85	0.034	***	N.S.	N.S.
Heart	1.36	1.33	1.34	1.52	1.57	1.48	0.018	***	N.S.	N.S.
Lung	1.73	1.62	1.64	1.74	1.86	1.75	0.179	**	*	*
Spleen	0.87	0.92	0.91	0.98	1.12	1.05	0.159	***	N.S.	N.S.

Abbreviation: N.S, Nonsignificant.

^a^ Control.

^b^ 5% coconut oil + 5% olive oil.

^c^ 10% olive oil.

^d^ Body weight.

^e^ Average daily gain.

^f^ Standard error of means.

* *p* < .05.

** *p* < .001.

*** *p* < .0001.

### 3.2 Effect of olive oil on the blood lipid profile

The blood lipid profiles of the rats that were fed with olive oil have been shown in Table 4. Determination of glutamic‐oxaloacetic transaminase (GOT) and glutamic pyruvic transaminase (GPT) are indicators for diagnosis of liver and heart diseases. These enzymes are released into the bloodstream upon disease or damage of body tissues, such as the liver or the heart tissues. Thus, GOT and GPT blood levels directly relate to the extent of tissue damage. After 4 and 8 weeks of feeding, GOT and GPT levels were not significantly affected by the different diets. However, the GOT and GPT levels tended to decrease with a further addition of oleic acid in the diet, as the olive100 group had the lowest GOT and GPT levels, followed by the olive50 group, and the control group. HDL cholesterols are regarded as good cholesterol, as HDL particles remove and return excess cholesterol molecules from the peripheral cells to the liver, and thus are inversely associated with a CVD risk. After 8 weeks, HDL‐C significantly increased with the further addition of olive oil, as the olive100 group exhibited the highest HDL‐C, followed by the olive50 group, and the CON group. Noteworthy, HDL‐C levels increased with the addition of olive oil after 4 weeks of feeding, but this increase failed to attain a statistical significance. Total cholesterol (TC) in both feeding periods was unaffected by different diets. High plasma triglycerides (TG) levels exhibit risk factors, including insulin resistance that may affect susceptibility to atherosclerosis and cardiovascular risk. Significantly low TG levels were observed at both 4 and 8 weeks of feeding in rats of the olive100 group, followed by the olive50 group, and the CON group.

**TABLE 4:** Plasma lipid profile of experimental animals that were fed with olive oil

Items	Treatments						*SEM* ^h^	Effects		
	4 weeks			8 weeks				Period(P)	Oil(O)	P × O
	CON^a^	Olive50^b^	Olive100^c^	CON^a^	Olive50^b^	Olive100^c^				
GOT^d^(IU/L)	86.94	79.45	72.93	73.2	68.77	62.12	2.259	*	N.S.	N.S.
GPT^e^(IU/L)	24.29	24.99	25.65	41.15	36.55	34.75	1.768	**	N.S.	N.S.
HDL^f^(mg/dl)	23.1	24.1	25.18	30.73	32.78	38.02	0.819	***	*	**
Total Cholesterol(mg/dl)	75.22	75.8	77.54	105.81	105.72	102.98	2.759	***	N.S.	N.S.
TG^g^(mg/dl)	104.6	81.64	72.65	181.45	165.41	154.58	8.279	***	N.S.	N.S.

Abbreviation: N.S, Nonsignificant.

^a^ Control.

^b^ 5% coconut oil + 5% olive oil.

^c^ 10% olive oil.

^d^ Glutamic‐oxaloacetic transaminase.

^e^ Glutamic pyruvic transaminase.

^f^ High‐density lipoprotein cholesterol.

^g^ Triglycerides.

^h^ Standard error of means.

* *p* < .05.

** *p* < .001.

*** *p* < .0001.

### 3.3 Fatty acid composition in blood plasma

22 fatty acids were detected in the blood plasma of the experimental rats, as shown in Table 5. SFAs, such as lauric acid (C12:0) and myristic acid (C14:0), were found to be significantly higher at 4 and 8 weeks in the blood plasma of experimental animals that were fed with 10% coconut oil. Stearic acid (C18:0), which is another SFA, was found to be significantly lower in experimental animals that were fed with 10% coconut oil at 4 and 8 weeks of the feeding period. MUFAs, such as myristoleic acid (C14:1) and palmitoleic acid (C16:1), were significantly higher in the experimental animals that were fed with 10% coconut oil; meanwhile, oleic acid (C18:1 *n*−9) was significantly higher in experimental animals that were fed with 10% olive oil—at 4 and 8 weeks for both comparisons. PUFAs, such as linolenic (ALA, C18:3 *n*−3), arachidonic (ARA, C20:4 *n*−6), eicosapentaenoic (EPA, C20:5 *n*−3), and docosahexaenoic (DHA, C22:6 *n*−3), were significantly increased, after both 4 and 8 weeks of feeding, in groups that were fed with 10% olive oil, followed by the olive50 group, and the control group. Linoleic acid (LA, C18:2 *n*−6), although insignificantly affected, tended to increase in the olive100 and the olive50 groups.

**TABLE 5:** Plasma fatty acid composition (mg/100 ml) of experimental rats from different treatment groups after 4 and 8 weeks of feeding

Fatty acid composition (g/100 ml) plasma	Treatments						*SEM* ^d^	Effects		
	4 weeks			8 weeks						
	CON^a^	Olive50^b^	Olive100^c^	CON^a^	Olive50^b^	Olive100^c^		Period (P)	Oil (O)	P × O
C_12:0 lauric acid_	3.72	1.28	0.39	4.83	1.18	0.51	0.25	N.S.	***	N.S.
C_14:0 myristic acid_	8.83	4.85	3.31	12.99	7.95	4.40	0.507	***	***	N.S.
C_14:1 myristoleic acid_	0.45	0.21	0.19	0.75	0.36	0.15	0.035	**	***	*
C_16:0 palmitic acid_	86.76	88.13	104.52	108.96	143.38	132.35	4.948	**	N.S.	N.S.
C_16:1 palmitoleic acid_	15.56	10.83	11.11	32.99	25.81	18.26	1.377	***	**	N.S.
C_18:0 stearic acid_	34.60	42.85	55.08	44.34	65.06	62.50	2.623	**	**	N.S.
C_18:1_ *_n_*_−9 oleic acid_	96.76	117.86	146.38	136.11	256.35	275.20	12.104	***	***	*
C_18:2_ *_n_*_−6 linoleic acid_	40.70	42.45	47.60	32.73	44.95	43.22	1.794	N.S.	N.S.	N.S.
C_18:3_ *_n_*_−3 linolenic acid_	0.91	1.04	1.28	0.54	0.74	0.82	0.052	***	*	N.S.
C_20:0 arachidic acid_	0.34	0.65	0.45	0.44	0.61	0.43	0.038	N.S.	*	N.S.
C_20:1_	0.68	0.97	1.13	1.46	2.71	3.01	0.153	***	**	N.S.
C_20:2_	0.21	0.25	0.21	0.34	0.47	0.48	0.024	***	N.S.	N.S.
C_20:3_	4.53	5.54	5.49	14.81	15.80	12.09	0.757	***	N.S.	N.S.
C_20:4 arachidonic acid_	89.32	112.25	150.76	89.80	134.53	137.00	5.184	N.S.	***	N.S.
C_20:5 eicosapentaenoic acid_	0.64	0.66	1.39	0.48	1.06	1.36	0.074	N.S.	***	N.S.
C_22:0_	0.21	0.28	0.13	1.28	0.74	0.34	0.081	***	**	*
C_22:1_	2.84	4.19	2.91	2.82	3.05	3.23	0.219	N.S.	N.S.	N.S.
C_24:0_	0.31	0.62	0.60	0.50	0.74	0.79	0.055	N.S.	*	N.S.
C_24:1_	0.22	0.41	0.48	0.27	0.58	0.95	0.071	N.S.	*	N.S.
C_22:6 docosahexaenoic acid_	9.78	13.79	19.83	8.40	15.70	17.80	0.742	N.S.	***	N.S.
Total SFA	134.77	138.66	164.48	173.34	219.66	201.32				
Total MUFA	116.51	134.47	162.20	174.40	288.86	300.80				
Total PUFA	146.09	175.98	226.56	147.10	213.25	212.77				
Total	397.37	449.11	553.24	494.84	721.77	714.89				

Abbreviation: N.S., Nonsignificant.

^a^ Control.

^b^ 5% coconut oil + 5% olive oil.

^c^ 10% olive oil.

^d^ Standard error of means.

* *p* < .05.

** *p* < .001.

*** *p* < .0001.

### 3.4 Fatty acid composition in adipose tissue

Only 13 fatty acids were detected in the adipose tissue of the experimental rats that were fed with olive oil, as shown in Table 6. After 4 and 8 weeks into the feeding period, SFAs, such as C12:0, C14:0, palmitic acid (C16:0), and C18:0, were significantly lower in the adipose tissue of the experimental animals that were fed with 10% olive oil. MUFAs, such as C14:1 and C16:1, were significantly higher in the adipose tissue of experimental animals that were fed with 10% coconut oil and those of the olive50 group (fed with 5% coconut oil + 5% olive oil). Further, oleic acid was significantly higher in the adipose tissue of experimental animals in the olive50 group and those that were fed with 10% olive oil—at both 4 and 8 weeks into the feeding period. After 4 weeks into the feeding period, LA was significantly higher in the experimental animals that were fed with 10% olive oil, which is comparable to that of the control diet, while ALA and ARA were significantly higher in experimental animals that were fed only with 10% olive oil. After 8 weeks of feeding, LA, ALA, and ARA were significantly higher in experimental animals that were fed with 10% olive oil. Other PUFAs, such as EPA and DHA, were not detected in the adipose tissue of experimental animals.

**TABLE 6:** Adipose tissue fatty acid composition (mg/100 ml) of experimental rats from different treatment groups after 4 and 8 weeks of feeding

Fatty acid composition (mg/100 g) plasma	Treatments						*SEM* ^a^	Effects		
	4 weeks			8 weeks						
	CON^a^	Olive50^b^	Olive100^c^	CON^a^	Olive50^b^	Olive100^c^		Period (P)	Oil (O)	P × O
C_12:0 lauric acid_	8.97	6.62	2.35	11.87	6.9	1.02	0.501	**	***	***
C_14:0 myristic acid_	6.4	4.94	2.69	8.1	4.98	1.67	0.289	*	***	***
C_14:1 myristoleic acid_	0.34	0.25	0.16	0.44	0.22	0.1	0.016	N.S.	***	***
C_16:0 palmitic acid_	25.19	23.58	22.52	25.23	22.46	19.57	0.284	***	***	***
C_16:1 palmitoleic acid_	7.35	6.33	5.97	8.63	6.16	4.53	0.186	N.S.	***	***
C_18:0 stearic acid_	2.54	2.46	2.62	1.99	2.06	2.27	0.037	***	***	N.S.
C_18:1_ *_n_*_−9 oleic acid_	36.43	44.82	51.9	33.28	47.16	60.65	1.23	***	***	***
C_18:2_ *_n_*_−6 linoleic acid_	6.69	5.51	6.54	3.62	3.84	4.45	0.186	***	**	*
C_18:3_ *_n_*_−3 linolenic acid_	0.25	0.23	0.29	0.09	0.16	0.24	0.011	***	***	**
C_20:0 arachidic acid_	0.02	0	0.02	0	0.01	0.05	0.003	N.S.	***	***
C_20:1_	0.18	0.23	0.25	0.12	0.2	0.27	0.008	*	***	*
C_20:3_	0.02	0.02	0.04	0.01	0.07	0.08	0.005	***	***	**
C_20:4 arachidonic acid_	0.05	0.05	0.06	0.03	0.07	0.07	0.005	N.S.	*	N.S.
Total SFA	43.12	37.6	30.2	47.19	36.41	24.58				
Total MUFA	44.3	51.63	58.28	42.47	53.74	65.55				
Total PUFA	7.01	5.81	6.93	3.75	4.14	4.84				
Total	94.43	95.04	95.41	93.41	94.29	94.97				

Abbreviation: N.S., Nonsignificant.

^a^ Control.

^b^ 5% coconut oil + 5% olive oil.

^c^ 10% olive oil.

^d^ Standard error of means.

* *p* < .05.

** *p* < .001.

*** *p* < .0001.

## 4 DISCUSSION

Cardiovascular disease (CVD) is globally the number one cause of death and a strong risk factor to CVD is the fats present in the human diet. In Korea, the Hanwoo industry has been criticized over the past decade due to the association of saturated fat to obesity and cardiovascular disease. Further, the Hanwoo beef is known to contain a high level of fat. Typically, the main SFAs in the IMF of beef are palmitic acid (C16:0) and stearic acid (C18:0) (Frank et al., 2016; Hwang & Joo, 2016, 2017), and the presence of these SFAs in beef is the main cause of health concerns and associations with CVD and obesity (Bingham et al., 2002). However, these SFAs only comprise slightly < 50% of the total lipid composition. The Hanwoo beef is highly marbled and contains an abundant amount of MUFA, such as oleic acid (Cho et al., 2005; Hwang & Joo, 2016, 2017; Jung, Hwang, & Joo, 2015, 2016), which has been described to reduce the risk of CVD by reducing blood lipids, primarily cholesterol. To provide a new and current evaluation of the effects of oleic acid on health‐related parameters, we evaluated the effects of oleic acid in olive oil on growth performance, blood lipid profile, and fatty acid composition in the blood plasma and the adipose tissue of rats.

### 4.1 Growth performance and serum biochemical profile

The final body weight of the experimental animals was significantly affected by the intake of oleic acid. The experimental animals that were fed with 10% olive oil exhibited the lowest final body weight, signifying that oleic acid can be associated with weight loss. This is consistent with a study by Liu et al. (2016), in which diets that were high in MUFA exhibited a greater oxidation rate, which consequently reduced the body weight. The comparable loss in the final body weight of experimental animals that were fed with 10% coconut oil to animals that were fed with 10% olive oil may be due to other fatty acids present in the diet. For instance, the control diet (10% coconut oil) contained a significantly high amount of lauric acid (5.14 mg/100 g), which is a medium‐chain fatty acid; further, fatty acids vary with the number of their carbons, which could lead to differences in their absorption, transportation, and destination (Papamandjaris, MacDougall, & Jones, 1998). MCFAs, such as lauric acid, are rapidly oxidized due to ease of absorption and transportation compared to those of the long‐chain fatty acids (LCFAs). The metabolic route that accompanies the rapid oxidation of MCFAs promotes satiety and increases energy expenditure, which may lead to a weight control. Thus, the loss of body weight in the control group may be due to the MCFA contents, and the loss of body weight in the experimental animals that were fed with 10% olive oil may be due to the high oleic acid content. In contrast, although the olive50 diet (5% coconut oil + 5% olive oil) exhibited a high oleic acid content (C18:1 *n*−9 = 4,634 mg/100 g), which contributed to weight loss, it also exhibited a high amount of total SFA (4,881 mg/100 g) and a low amount of MCFA (2,487 mg/100 g), which may have hindered the effect of oleic acid on weight loss of the experimental animals. Thus, this may explain the increased final body weight of the experimental animals that were fed with the olive50 diet.

GOT and GPT enzymes are released into the bloodstream upon disease or damage of body tissues, such as tissues of the liver or the heart; thus, these enzyme levels are used as indicators to diagnose liver and heart diseases. In this study, GOT and GPT levels were not significantly affected by different diets after 4 and 8 weeks of feeding, although GOT and GPT levels tended to decrease with the addition of oleic acid, with the lowest level observed in the olive100 group, followed by the olive50 group, and the control group. Dietary oleic acid in olive oil exhibited a potential attenuation effect—though statistically insignificant—on GOT and GPT levels in the blood plasma. HDL‐cholesterols are regarded as good cholesterol, since HDL particles remove and return excess cholesterol molecules from the peripheral cells to the liver, and thus are inversely associated with a CVD risk. After 4 weeks of feeding, HDL‐C increased at statistically insignificant levels with the addition of olive oil. After 8 weeks of feeding, HDL‐C significantly increased with the addition of olive oil, which suggests that oleic acid can increase HDL‐C after a long period of consumption and may lower risk of CVD. Moreover, significantly low TG levels were observed at 4 and 8 weeks into the feeding period in experimental animals that were fed with 10% olive oil, which suggests that oleic acid may play a great role in reducing triglycerides in the bloodstream with short‐ and long‐term OA consumption. Thus, OA can potentially reduce risk factors, such as insulin resistance, which may affect susceptibility to atherosclerosis and CVD.

### 4.2 Fatty acid composition in blood plasma and adipose tissue

Fatty acids of tissue and blood are mainly derived from diet; hence, dietary intake considerably determines the fatty acid composition in the adipose tissue and blood. This study showed that the intake of dietary oleic acid in olive oil significantly affected the fatty acid composition of the blood plasma. Intake of 10% olive oil reduced levels of lauric and myristic acids in the blood plasma of experimental rats due to the low lauric and myristic acid content in the diet. SFA—stearic acid, MUFA—OA, *n*−*3* PUFAs ALA, EPA, and DHA were found to be significantly increased in the blood plasma of experimental rats that were fed with 10% olive oil. The 10% olive oil diet contained elevated stearic acid levels, most of which may not be converted to OA in the adipose tissue and thus was carried to the blood plasma. In addition, the diet with 10% olive oil also exhibited a high LA content, and LA is further converted to stearic acid, which may have contributed to the stearic acid content in the blood plasma. Stearic acid, although an SFA, exhibited no effects on the total serum cholesterol concentration, which is a cause of an increased CVD risk (Hegsted, Mcgandy, Myers, & Stare, 1965; Keys, Anderson, & Grande, 1965). Olive oil is a good source of OA, and the diet with 10% olive oil exhibited a high OA content. Since it was shown in this study that plasma and dietary OA levels were only weakly correlated (Mensink, Zock, Kester, & Katan, 2003), OA content was found to be elevated in the blood plasma of rats that were fed with 10% olive oil, but blood plasma OA may also reduce a CVD risk. Other fatty acids that were shown to be at high levels in the diet with 10% olive oil were LA and ALA, and these FAs, along with OA, are competitive substrates for the same order of desaturation enzymes. In this study, *n*−*6* PUFA ARA increased with increased production levels of the metabolism of the high dietary LA content in the diet with 10% olive oil. The *n*−*3* PUFAs EPA and DHA also increased as the product levels of the metabolism of the high dietary ALA content increased in the diet with 10% olive oil. Similar results were observed in the blood plasma of experimental rats that were fed with 5% olive oil—only with lower FA content than for rats that were fed with 10% olive oil. The fatty acid composition in the blood plasma of the experimental rats that were fed with 10% olive oil was reflected by the dietary fatty acid composition. The results of this study suggest that the intake of high oleic acid in an olive oil‐supplemented diet may enhance the levels of oleic acid, ARA, and the omega‐3 fatty acids EPA and DHA in the blood plasma. The OA content in the adipose tissue increased after the consumption of diet with 10% olive oil. It is well established that stearic acid is converted into oleic acid in the adipose tissue. High dietary stearic acid content was converted to oleic acid and used to store lipids in the adipose tissue. PUFAs, such as LA and ALA, were found in low amounts, but olive oil intake led to a significantly higher content than those in the controls. Other PUFAs, such as EPA and DHA, were not detected in the adipose tissue of experimental rats. The low PUFA levels detected in the adipose tissue implied that olive oil‐based dietary OA was utilized and transported in the bloodstream and minimally used in lipid synthesis in adipose tissue, thus improving the blood lipid profile and potentially reducing a CVD risk.

In conclusion, our findings suggest that dietary intake of high oleic acid may enhance omega‐3 fatty acids in the blood plasma of rats and may have attenuation effects on GOT and GPT levels in the bloodstream, as well as the positive effect of reducing risk of CVD—as evidenced by weight loss, increased HDL‐C, and decreased TG levels.

## 5 ETHICS APPROVAL

All experimental procedures complied with the NIH Guidelines for Care and Use of Laboratory Animals.

## CONFLICT OF INTEREST

The authors have no potential conflict of interest relevant to this article to report.
